# Substance abuse and intimate partner violence: treatment considerations

**DOI:** 10.1186/1747-597X-1-24

**Published:** 2006-08-22

**Authors:** Keith C Klostermann

**Affiliations:** 1 RTI International, 3040 Cornwallis Road, Hobbs Building, Research Triangle Park, NC 27709-2194, USA

## Abstract

Given the increased use of marital- and family-based treatments as part of treatment for alcoholism and other drug disorders, providers are increasingly faced with the challenge of addressing intimate partner violence among their patients and their intimate partners. Yet, effective options for clinicians who confront this issue are extremely limited. While the typical response of providers is to refer these cases to some form of batterers' treatment, three fundamental concerns make this strategy problematic: (1) most of the agencies that provide batterers' treatment only accept individuals who are legally mandated to complete their programs; (2) among programs that do accept nonmandated patients, most substance-abusing patients do not accept such referrals or drop out early in the treatment process; and (3) available evidence suggests these programs may not be effective in reducing intimate partner violence. Given these very significant concerns with the current referral approach, coupled with the high incidence of IPV among individuals entering substance abuse treatment, providers need to develop strategies for addressing IPV that can be incorporated and integrated into their base intervention packages.

## Substance abuse and intimate partner violence: treatment considerations

While historically considered a private family matter, intimate partner violence (IPV) has more recently been conceptualized as a widespread public health concern, requiring the attention of both the treatment community and criminal justice system. In fact, representative surveys of couples, which include less severe instances of aggression, such as single occurrences of pushing or slapping one's partner, suggest rates of 15% to 20% annually for any husband-to-wife violence [1,2]. Yet, these estimates are dwarfed in comparison to those observed among married or cohabiting substance-abusing patients entering substance abuse treatment. More specifically, studies conducted over the last decade have consistently revealed that roughly 60% of substance-abusing men with intimate partners report at least one instance of IPV during the year prior to program entry. Given the increased use of family-involved assessments and interventions in substance abuse treatment programs, providers are increasingly faced with the challenge of addressing this complex clinical issue.

Unfortunately, effective treatment options for providers who must deal with this issue are limited. To date, the typical answer has been for providers to refer these cases to agencies specializing in batterers' treatment. However, there are three fundamental problems with this strategy. ***First***, many batterers' treatment programs will only accept individuals who are specifically mandated by the legal community to participate in IPV treatment. Yet, most patients in substance abuse treatment settings are not required to attend a batterers' program; in fact, a large majority of substance-abusing patients are not identified as having engaged in IPV or are only so identified after lengthy or careful assessment while receiving treatment for substance abuse. ***Second***, in those instances in which batterers' programs will accept referrals of nonmandated substance-abusing patients, the vast majority of these patients typically either do not attend the batterers' intervention or drop out early in the treatment process. ***Third***, and perhaps most important, results of a recent meta-analytic review indicate that batterers' intervention programs are largely ineffective in reducing partner aggression. Given these very significant problems with the current referral approach, substance abuse treatment programs need to develop strategies for addressing IPV that can be incorporated into their intervention packages.

Thus, the purpose of this article is to explore what is known about IPV, with an emphasis on the association between substance abuse and IPV. Current strategies for addressing IPV in substance abuse treatment settings will also be reviewed. Finally, recommendations for treatment directions to treat IPV among alcoholic and drug-abusing patients and their partners are provided.

### Defining IPV

A number of theories have been proposed to explain the factors that cause and contribute to IPV. From a feminist perspective, IPV is viewed as a matter of control and has roots in the historical traditions of male dominance in intimate relationships (e.g., marriage) [3,4]. Family violence theory views IPV as a matter of conflict, produced from the daily stresses of life that often result in conflict. These conflicts, in turn, have the potential to escalate into violence [5].

IPV encompasses a wide range of physically aggressive behaviors between partners that vary greatly along such dimensions as (a) type and severity of aggression (e.g., a push versus an injury-inducing beating), (b) frequency (e.g., a single shove versus repeated shoving over an extended time frame), and (c) emotional and physical impact (i.e., aggression that induces fear) [6]. Along these lines, Johnson [7] developed a model of IPV based on the control context within the relationship. More specifically, Johnson describes three types of IPV that appear to be conceptually and etiologically distinct. The first, *Intimate Terrorism*, is distinguished by severe male-to-female physical aggression (e.g., punching, threatening with weapons), with less severe female-to-male violence occurring during these episodes as a manner of self-defense (i.e., Violent Resistance). For the female partner, this severe type of violence is usually accompanied by an increased likelihood of physical injury and increased fear of the male partner. In instances of Intimate Terrorism, the aggression serves the purpose of dominating and controlling the partner, which is typically displayed through a wide range of power and control tactics, including violence [8]. As noted above, the second type, *Violent Resistance*, is characterized by violence that occurs in response to a partner's violent and controlling behavior (e.g., Intimate Terrorism). In these cases, the resistor is violent, but not controlling. The last type, *Situational Couple Violence*, is characterized by bidirectional partner aggression (i.e., violence that may be initiated by either partner), which is mild to moderate in severity, and typically occurs as a reaction as a conflict escalates. In general, Situational Couple Violence is not used as a form of control and is also less likely to cause fear in or endanger the female partner. In general, Situational Couple Violence is likely to be akin to violence reported in the general population surveys, whereas Intimate Terrorism more closely resembles the violence typically found in clinical samples. The primary distinctions among these three types of violence are related to patterns of power and control.

Much of the debate about IPV is focused on Intimate Terrorism, despite the fact that most partners who report and enter treatment for IPV engage in violence that more closely resembles Situational Couple Violence. This seems to be the case for violent couples in which a partner enters substance abuse treatment; the vast majority of these couples (i.e., over 95%) report episodes of partner aggression that are similar to descriptions of common couple violence than patriarchal terrorism [9].

### Prevalence of IPV

Depending on the definition of violence used, as well as the context in which it is examined, estimates of physical aggression between partners may vary widely. The Department of Justice estimates that roughly 1,500 instances of homicide and manslaughter between intimate partners occur annually, with more than 1,200 of these involving women as victims [10]. In addition, approximately 250,000 emergency room visits in the U.S. each year involve a victim of IPV. Moreover, the National Crime Victim Survey [11] reports that nearly 1 million women are victims of IPV annually. Findings of numerous studies indicate one out of every eight husbands engages in some form of physical aggressive behavior, including less severe episodes of aggression (e.g., single episodes of pushing or slapping) against his intimate partner [1,2]. Interestingly, it appears that women perpetrate physical aggression in their intimate relationships at similar or slightly higher rates than men [12]; however, the consequences of male-to-female physical aggression appear to be significantly greater on the female partners [13].

However, it is worth acknowledging that there is much debate around the incidence and prevalence of men's and women's IPV. In fact, there has been much disagreement among researchers about definitions, methods used, and the results concerning the direction and impact of violence between men and women in intimate relationships [14]. More specifically, several authors [14-18] have argued that quantitative act-based measures (including the Conflict Tactics Scale; CTS) undercount men's perpetration of IPV, and thus, do not provide an accurate reflection of true levels of IPV. These researchers argue that the use of a narrow, act-based approach to defining and measuring violence is more likely to find symmetry between men and women in their reports of violence.

### Substance use, intoxication, and IPV: the debate

The occurrence of violence between intimate partners is thought to be the result of multiple interacting factors (e.g., contextual, social, biological, psychological, and personality), which exert their influence at different times, under different circumstances, acting in a probabilistic fashion [19]. Of the various components that have been identified in conceptual and predictive models of IPV, alcohol use is among the most controversial and widely debated. While there is agreement that those who engage in IPV often drink and that intoxication often accompanies violence, there is considerable debate as to whether or not alcohol use simply covaries with partner violence, is inherently facilitative or a contributing cause of IPV, or is simply an "excuse" for aggression. Thus, this debate has important treatment implications. More precisely, if intoxication is causally linked to IPV, it would follow that interventions that are successful in reducing drinking could reduce the occurrence of partner violence.

### Treatment options for IPV among substance-abusing patients

Unfortunately, there is a lack of agreement about the best treatments for IPV among patients entering substance abuse treatment. Comprehensive evaluations of different types of interventions for IPV are only now beginning to emerge. In the following, I describe some of the typical responses to IPV by substance abuse treatment programs, as well as less commonly used approaches, and highlight the evidence for their respective effectiveness.

#### Treatment-As-Usual (TAU): standard substance abuse treatment

Given the increase in prevalence of IPV among men seeking substance abuse treatment, it seems substance abuse treatment programs may represent a critical point of entry for addressing IPV. Yet, surveys of substance abuse treatment agencies reveal that referral to domestic violence intervention programs is rare [20,21]. In fact, individuals entering alcoholism treatment are typically not assessed for IPV or, if they are, the assessments themselves are inadequate [9].

Nonetheless, if alcohol use is causally linked to IPV, standard treatment for substance abuse might be an effective intervention for IPV; results of recent studies provide support for this contention. For example, O'Farrell et al. [22] conducted a study examining IPV among alcoholic men (*N *= 301) entering a typical outpatient substance abuse treatment program, in which IPV was not the focus of treatment. In the year before treatment, 56% of the alcoholic patients perpetrated violence toward their female partners, compared with a rate of 14% in a demographically matched nonalcoholic comparison sample. In the year after treatment, IPV decreased to 25% among all treated patients, but was only 15% among remitted alcoholics and 32% among relapsed patients.

While there is a paucity of research in this area with female alcoholic clients, available results are similar to those obtained with male alcoholic patients. For example, Stuart et al. [23] examined the effect of intensive alcoholism outpatient treatment on IPV perpetration and victimization among female patients. Results indicated a decrease in both the prevalence and frequency of partner violence after treatment. Moreover, women who relapsed during the 1-year posttreatment follow-up period were more likely to perpetrate IPV than those women who had not relapsed.

Interestingly, IPV does appear to decrease as a result of standard alcoholism treatment, particularly among patients who did not relapse in the posttreatment period. These findings support the notion that clients who have problems with alcohol should receive substance abuse treatment as a component of an overall intervention for IPV. However, the major drawback to this approach in addressing IPV is that the violence reductions appear to rely on alcohol abstinence. Other factors (e.g., conflict resolution skills, partner responses to patients' relapses, etc.) that may contribute to IPV are typically ignored or not addressed as part of the standard substance abuse treatment. Given the relapse rates typically reported for patients after substance abuse treatment, plus the many-fold increase in the likelihood of IPV on days of alcohol use after treatment completion, standard substance abuse treatment may best be viewed as a necessary, but not sufficient, intervention for patients seeking help for alcoholism who have also engaged in IPV.

#### Referral to domestic violence intervention programs

As previously noted, it could be argued that a reasonable approach would be to train substance abuse treatment programs to assess and accurately identify incoming patients who have engaged in IPV and then refer those patients to domestic violence intervention programs. However, two critical issues make the referral strategy approach more problematic than it may appear. First, many domestic violence interventions are considered most appropriate for perpetrators of IPV mandated by the criminal justice system in which a swift and certain court response to violations is implemented [24,25]. Thus, the potential for legal ramifications serves as a powerful motivator for clients to participate in these programs. In contrast, patients entering substance abuse treatment who perpetrate IPV are rarely mandated by the criminal justice system to also participate in some form of domestic violence intervention programs as part of their overall treatment plan. A review of records across multiple community-based substance abuse treatment programs revealed that less than 2% of patients were mandated to also participate in a domestic violence intervention program. Although most domestic violence programs admit nonmandated patients, available evidence suggests that few alcohol-dependent patients accept a referral to these programs, or those that do typically drop out very early in the course of the intervention [26]. Simply stated, this very low level of engagement and participation is likely due to the fact that very few alcohol-dependent patients are coerced by the criminal justice system to participate in these batterer intervention programs. Additionally, linkages between domestic violence and substance abuse treatment programs are usually very poor, and thus, little effort is made to monitor and coordinate, effectively, the referral process.

### IPV treatment options

The most common model for treating IPV in community settings is referred to as Gender Specific Treatment (GST) [27]. This model was originally developed as part of the Duluth Domestic Abuse Intervention Project in Minnesota and is also known as the Duluth Model. These programs emphasize two interrelated themes about IPV: (a) it is a purposeful and systematic behavior by men to exert power and control over their partners, and (b) it results from the patriarchal structure of society. According to the Duluth Model, male partners must: (1) take full responsibility for occurrences of IPV and for stopping such abuse, (2) acknowledge and recognize their need for power and control at the familial and societal level, and (3) accept that their abusive beliefs about power and control perpetuate aggression in the home. The treatment delivery format is typically male-only groups, which are used to emphasize men's sole responsibility for episodes of IPV [28]. In general, these domestic violence intervention programs emphasize accountability and safety for the partner. Additionally, in many programs, accountability is seen as possible only when there is certainty that the criminal justice system will impose swift, consistent, and meaningful sanctions [25].

Yet, the evidence for the effectiveness of domestic violence intervention programs in reducing or eliminating IPV has been mixed. Results of a recent meta-analytic review revealed little or no effects for these programs [29], a conclusion that has been supported by other recently completed experimental studies. Gondolf [24], however, noted several limitations of these studies, and based on the results of a multisite evaluation of batterer treatment programs, he concluded these programs have moderate treatment effects. Despite the questionable effectiveness of these programs, it could be argued that it is better to provide some form of focused intervention, than to do nothing. This view assumes the intervention, even an ineffective one, would at least do no harm. However, there are serious implications to this perspective. As an example, suppose a violent male completes a domestic violence program. If the patient's partner incorrectly believes the program has been effective in treating her partner (i.e., reduced or eliminated the likelihood of IPV), she may behave differently based on this assumption (e.g., she may return home if she has left, she may engage in an emotionally-charged argument that she might otherwise have avoided). Along these lines, if there has been no attempt to address alcohol use, any of the "lessons learned" may be negated during episodes of drinking. Thus, in this case, participation in an ineffective domestic violence program or even one that is marginally effective, but has not addressed the role of alcohol use, may actually *increase *the likelihood of potential harm. Results of a study by Gondolf [30] are consistent with this contention. In that study, more than 6,000 women leaving battered women's shelters were queried as to whether they intended to return to their abusive partners or leave them. The strongest predictor of women's decisions was whether or not their partners participated in some form of domestic violence treatment. More specifically, if the male partners were involved in domestic violence treatment, 53% of the wives planned to return to them; if the male partners were not participating in domestic violence treatment, only 19% of the women planned to return.

#### Conjoint therapy

Partner-involved therapies are among the most controversial and widely debated treatment approaches for IPV. In much of the IPV literature, marital and family therapies for IPV are typically viewed as inappropriate, ineffective, ethically questionable, and potentially dangerous [25]. The controversy is based on the following assumptions: (a) conjoint therapy models highlight participants' shared responsibility for the behavior, with the victim assuming she is at least partially responsible for her partner's violence, and thus, the abuser is able to conclude he is not fully responsible for his own aggressive behavior; and (b) conjoint counseling encourages honest and open disclosure, which could facilitate conflict in therapy sessions that could escalate to violence outside of therapy. Consequently, most states have implemented standards and guidelines that discourage the use of or prohibit funding to programs that offer couples or family therapy as an intervention for IPV [31,32].

Alternatively, some researchers have recognized the potential advantages that partner-involved treatments may have for couples who engage in IPV [33]. First, a more comprehensive evaluation of the level and severity of IPV can be obtained because both partners are providing information on situations in which reports and descriptions of IPV are often discrepant [1]. Conjoint therapy also provides a safer environment for partners to discuss high conflict and emotionally-charged topics; these discussions can also be delayed until the partners meet with the therapist, which can help them avoid such topics at home until they have the skills necessary to discuss such issues constructively. Based on previous research that partner aggression most often occurs in the context of arguments between partners [34] and is often mutual and bidirectional [35], addressing the interactional nature of the partner aggression may reduce its frequency by altering the interaction patterns that precede it. Since relationship distress is a powerful predictor of partner aggression [36], improvements in a couple's functioning (a primary goal of conjoint treatment) should reduce the likelihood of IPV.

Interestingly, in the three studies that compared gender-specific group therapy approaches to conjoint treatment with partner-aggressive men and their partners, both types of treatment led to IPV reductions, but no group differences in levels of IPV were found [37-39]. Couples participating in these studies were interested in remaining together and were willing to engage in conjoint therapy; as such, these dyads may be dissimilar from couples in which partners are entering domestic violence programs. However, it's worth noting that these couples may not be so different in important respects from couples in which a partner is entering substance abuse treatment.

### Behavioral Couples Therapy (BCT) for alcoholism and substance abuse: effects on IPV

A couples-based treatment for substance abuse that has extensive empirical support for its clinical and cost effectiveness is BCT [40]. BCT is a partner-involved treatment for substance abuse which teaches skills that promote partner support for abstinence and emphasizes the resolution of common relationship problems. For a review, see Klostermann, Fals-Stewart, et al., 2005 [41].

In regard to IPV, nonsubstance-abusing partners are taught coping skills to increase safety when faced with a situation where the likelihood of IPV is heightened. More specifically, behaviors that reduce the likelihood of aggression when a partner is intoxicated (e.g., leaving the situation, avoiding conflictual and emotionally-laden discussion topics with an intoxicated partner) are emphasized. Thus, BCT is designed to reduce partner violence in these couples even when relapse occurs. In contrast to traditional individual treatment for substance abuse, BCT does not rely exclusively on abstinence as the mechanism of action for nonviolence.

A number of studies have examined the effects of BCT on IPV prevalence and frequency of IPV among substance-abusing men and their nonsubstance-abusing female partners. In these investigations, the type of violence reported by couples typically resembled Situational Couple Violence. O'Farrell, Murphy, Stephan, Fals-Stewart, and Murphy [42] replicated, with a large heterogeneous intent-to-treat sample, initial study findings of dramatically reduced male partner physical violence associated with abstinence after BCT [43]. In this study, IPV was examined pre and post BCT for 303 married or cohabiting male alcoholic patients; the study also included a demographically matched non-alcoholic comparison sample. Findings showed in the year prior to BCT, 60% of alcoholic patients had been violent toward their female partners, which was five times the comparison sample rate of 12%. In turn, in the year after BCT, violence decreased to 24% in the BCT group, but remained higher than the comparison group. Among remitted alcoholics after BCT, the rates of violence were reduced to 12%, identical to the comparison sample and less than half the rate among relapsed patients (30%). Results at the 24-month post BCT point revealed similar findings. Interestingly, Chase and colleagues [44] reported similar findings with a sample of married or cohabiting alcoholic women and their nonsubstance-abusing male partners who engaged in BCT.

Fals-Stewart, Kashdan, O'Farrell, and Birchler [45] examined changes in IPV among 80 married or cohabiting drug-abusing patients and their nonsubstance-abusing female partners randomly assigned to receive either BCT or individual treatment. While almost half of the couples in each condition reported male-to-female IPV during the year before treatment, the number reporting violence in the year after treatment was significantly lower for the BCT group (17%) compared to the individual treatment for the male partner only group (42%). Mediation analyses indicated BCT led to greater reductions in IPV because participation in BCT reduced drug use, drinking, and relationship problems to a greater extent than individual treatment.

Importantly, BCT is designed to reduce partner violence in these couples even when relapse occurs. In contrast to traditional individual treatment for substance abuse, BCT does not rely exclusively on abstinence as the primary mechanism of action for nonviolence.

Fals-Stewart [46] randomly assigned couples with an alcoholic male partner and recent history of IPV to one of three treatment conditions: (a) BCT, (b) individual-based alcoholism treatment for the male partner only, and (c) a psychoeducational attention control treatment for couples. During the year after treatment, the likelihoods of IPV on days of substance use for couples in the three conditions were compared. All of the treatments were equally effective in reducing male-to-female physical aggression on days in which the male partner did not drink. However, on days of male partner drinking, the likelihood of male-to-female physical aggression was significantly reduced (i.e., 51% lower on average) for couples who received BCT compared to the couples in the other conditions.

While these results are indeed impressive, there are two primary limitations of these investigations that make drawing more definitive conclusions difficult. First, the Chase et al. [44] and O'Farrell et al. [42] investigations were essentially pre-post designs (with the concomitant threats to validity) and used act-based measures. Second, the Fals-Stewart et al. [45] investigation did not recruit couples who engaged in IPV; these results were culled from a larger study of BCT for drug abuse that happened to include a proportion of couples who reported IPV in the year prior to study entry. Thus, the sizeable clinical effects observed for BCT, in terms of levels of IPV, coupled with the study design limitations noted, reveal the need for further study in this area.

## Conclusion

Given the increased attention to IPV and the public's growing demand for action on the part of the legal and treatment communities to address this problem, it is unfortunate that results of studies examining the effectiveness of interventions for IPV have been mixed. While some studies indicate that batterer treatment programs are moderately effective [24], the findings of a recently completed meta-analytic review reveals little or no effects for these programs [29], a conclusion that is consistent with several recently completed experimental studies [47]. These findings raise important questions about the usual response to IPV by clinicians and members of the criminal justice system of mandating perpetrators to traditional domestic violence treatment. While it appears that treating alcohol use is an effective approach to reducing IPV, this is not a common strategy. In fact, there is limited clinical research describing approaches for addressing IPV and alcohol use, and the few approaches that have been recommended lack empirical support [48].

Despite the controversy surrounding conjoint treatments for IPV, carefully conceptualized and delivered couples treatment appears to be at least as effective as traditional treatment for IPV [49]. A conjoint treatment for alcoholism that has received extensive empirical support for its effectiveness is BCT. A series of studies have demonstrated the effects of BCT on reducing the prevalence and frequency of IPV among substance-abusing men and their nonsubstance-abusing female partners who have experienced low levels of violence [43,45,46]. Future investigations might assess the effectiveness of BCT in reducing alcohol use, relationship distress, and levels of IPV among a sample of men specifically identified as having substance abuse problems and who've perpetrated low levels of violence in their relationship. If BCT is not only effective at reducing alcohol use and relationship distress, but also levels of IPV, it would result in a research-supported integrated treatment manual that could easily be disseminated to community providers.

Given the mixed empirical support of current treatments, coupled with our ideas on improving existing treatments, it is now our responsibility to apply what we know about this complex problem to improve and develop new treatments [50]. The consequences of treatment failure are very salient in IPV research. Although well-intentioned, it is important to recognize that doing what we have been doing in most substance abuse treatment programs (e.g., standard substance abuse treatment without attention to IPV, referral to domestic violence programs with very high dropout rates and mixed IPV outcomes) is also potentially placing patients and their families at risk. Future studies may wish to further develop or examine new integrated IPV and substance abuse treatment models, including conjoint approaches. The evidence from studies of at least one conjoint therapy (BCT), where an IPV focus is combined with an abstinence focus, suggests there are some conditions where conjoint therapies can be a substantial improvement over conventional choices. Given the current direction of the field toward a "coordinated community response," substance abuse treatment programs may also wish to develop a strategic plan to address IPV, in terms of strengthening referral linkages to other providers or developing requisite expertise among program staff to treat partner violence. Finally, given the debate of sexual symmetry in IPV rates, we need a better understanding of IPV by female partners in their relationships, how much of it is defensive responding, how much of it is unidirectional versus interactional, and what is the best way to measure this phenomenon. Thus, there is a need for research focused on understanding and synthesizing the factors that have been identified as contributing to IPV and alcohol use and on treatments designed to address these contributing factors [48].

## Competing interests

The author declares that he has no competing interests.

## Authors' contributions

The author declares that he is the sole author of the article.
